# Practical application of opt-out recruitment methods in two health services research studies

**DOI:** 10.1186/s12874-017-0333-5

**Published:** 2017-04-14

**Authors:** Christopher J. Miller, James F. Burgess, Ellen P. Fischer, Deborah J. Hodges, Lindsay K. Belanger, Jessica M. Lipschitz, Siena R. Easley, Christopher J. Koenig, Regina L. Stanley, Jeffrey M. Pyne

**Affiliations:** 1 VA Boston Healthcare System Center for Healthcare Organization and Implementation Research and Harvard Medical School Department of Psychiatry, Boston, Massachusetts USA; 2 VA Boston Healthcare System Center for Healthcare Organization and Implementation Research and Boston University School of Public Health, Boston, Massachusetts USA; 3 Center for Mental Healthcare & Outcomes Research, Central Arkansas Veterans Healthcare System, and University of Arkansas for Medical Sciences Department of Psychiatry, Little Rock, Arkansas USA; 4 Central Arkansas Veterans Healthcare System and University of Arkansas for Medical Sciences, Little Rock, Arkansas USA; 5 San Francisco VA Health Care System and San Francisco State University Department of Communication Studies, San Francisco, California USA; 6 Central Arkansas Veterans Healthcare System, Little Rock, Arkansas USA; 7 Central Arkansas Veterans Healthcare System and University of Arkansas for Medical Sciences Department of Psychiatry, Little Rock, Arkansas USA

**Keywords:** Recruitment, Access, Health services, Opt-out

## Abstract

**Background:**

Participant recruitment is an ongoing challenge in health research. Recruitment may be especially difficult for studies of access to health care because, even among those who are in care, people using services least often also may be hardest to contact and recruit. Opt-out recruitment methods (in which potential participants are given the opportunity to decline further contact about the study (opt out) following an initial mailing, and are then contacted directly if they have not opted out within a specified period) can be used for such studies. However, there is a dearth of literature on the effort needed for effective opt-out recruitment.

**Methods:**

In this paper we describe opt-out recruitment procedures for two studies on access to health care within the U.S. Department of Veterans Affairs. We report resource requirements for recruitment efforts (number of opt-out packets mailed and number of phone calls made). We also compare the characteristics of study participants to potential participants via t-tests, Fisher’s exact tests, and chi-squared tests.

**Results:**

Recruitment rates for our two studies were 12 and 21%, respectively. Across multiple study sites, we had to send between 4.3 and 9.2 opt-out packets to recruit one participant. The number of phone calls required to arrive at a final status for each potentially eligible Veteran (i.e. study participation or the termination of recruitment efforts) were 2.9 and 6.1 in the two studies, respectively. Study participants differed as expected from the population of potentially eligible Veterans based on planned oversampling of certain subpopulations. The final samples of participants did not differ statistically from those who were mailed opt-out packets, with one exception: in one of our two studies, participants had higher rates of mental health service use in the past year than did those mailed opt-out packets (64 vs. 47%).

**Conclusions:**

Our results emphasize the practicality of using opt-out methods for studies of access to health care. Despite the benefits of these methods, opt-out alone may be insufficient to eliminate non-response bias on key variables. Researchers will need to balance considerations of sample representativeness and feasibility when designing studies investigating access to care.

**Electronic supplementary material:**

The online version of this article (doi:10.1186/s12874-017-0333-5) contains supplementary material, which is available to authorized users.

## Background

Participant recruitment is a significant challenge in health services and health-related research [1, 2]. It may be particularly challenging when a representative sample is desired, as is often the case in survey research, or when constructing purposeful samples with specific characteristics, as is often the case in qualitative research. Timely recruitment of such samples can be especially difficult for studies of access to health care because, even among those who are currently in care, people who use services least often also may be the hardest to contact and recruit. Some of the most common recruitment methods, like convenience sampling via self- or clinician-referral, may be least appropriate for such studies, as these sampling strategies are likely to draw primarily on frequent service users. This is problematic because data from this population may not capture the full range of challenges to access experienced by individuals who rarely use services.

Many recruitment methods are commonly used in health services research. These include—but are not limited to—snowball sampling, in which participants are asked to name other potential participants [3, 4]; and respondent-driven sampling, which combines snowball sampling with statistical weighting to account for non-random sampling [5–7]. Other studies have used advertisements in the form of postings, online ads, radio, or TV; or direct outreach, for example “cold-calling” potential participants [8]. Both opt-in and opt-out methods have also been used. Opt-in methods require potential participants to respond to an initial mailing before being contacted directly by study staff, while opt-out methods involve sending potential participants an informational packet about the study, giving them an opportunity to opt out (i.e., decline participation), and then contacting them only if they have not opted out within a predetermined period [3].

One key research component helps determine which of the above methods may be appropriate for any given study—namely, whether or not the study aims to draw from a pre-defined list of potential participants (a “sampling frame”). Such a sampling frame may, for example, be compiled from a healthcare system’s electronic medical record, or a health plan’s database of users. In such situations, direct outreach, opt-in and opt-out strategies are all technically feasible. However, following adoption of privacy legislation like the Health Insurance Portability and Accountability Act (HIPAA) in the United States [9], and the Data Protection Act in the United Kingdom [10], institutional review boards (IRBs) may restrict or prohibit use of opt-out methods in health-related research. Subsequent research has consistently demonstrated, however, that opt-out methods result in substantially higher recruitment rates than opt-in methods, and generate more representative samples [2, 11–19]. In this context, researchers often prefer opt-out methods, at least for low-risk studies [20, 21].

Published findings on the superiority of opt-out methods for recruitment are consistent with psychological and behavioral theories of motivation [22, 23], especially when opt-out methods are being compared to opt-in approaches. Opt-out methods may allow for more robust and unbiased recruitment by placing the burden for outreach on the research team rather than on potential participants or clinicians. Opt-in methods, in contrast, require potential participants to take the initiative by reaching out to study staff after receiving an initial mailing. That is something only individuals who are highly interested in the study are likely to do. In contrast, when opt-out approaches are used, only those potential participants who are highly disinterested in the study are likely to respond to the initial mailing to decline further contact (and thereby, study participation). Opt-out methods allow research staff to directly contact individuals who may be willing to participate, but not willing to take the initiative required for opt-in methods, maximizing chances of recruiting and obtaining information from this generally underrepresented subgroup of potential research participants. At the same time, by providing an opportunity for potential participants to decline further contact, opt-out methods may address concerns regarding intrusiveness and coercion.

Much of the existing literature on opt-out methods has been focused on clinical observational studies (e.g. [15]) or epidemiologic studies (e.g. [24]). Few studies described recruitment to health service-oriented studies (e.g. [12]), and none of the studies we located provided comprehensive data on the recruitment efforts and resources needed to obtain samples from administrative databases. In this paper, we therefore seek to enhance the literature base informing researchers on use of opt-out strategies by describing our experiences using these methods in two studies of access to mental health care among U.S. military Veterans (one featuring mixed quantitative and qualitative methods, and the other focusing on a quantitative telephone survey). In practical terms, we aimed to (1) compare our participant sample to the larger pool of potential participants mailed an opt-out letter, and (2) provide practical details on the resource demands (time and cost) required to apply our opt-out procedures. Researchers designing, budgeting, and executing these types of studies should be able to use our results to assist them in making more realistic assessments of sampling outcomes. Based on the literature reviewed above, our *a priori* hypothesis was that, across both studies described herein, the sample of study participants would be statistically similar to the larger pool of potential participants who were mailed an opt-out packet.

## Methods

The sampling frames for our two studies (referred to below as “Access” and “Tailoring”) were identified through administrative data from the U.S. Department of Veterans Affairs (VA) Corporate Data Warehouse (CDW), the national clinical administrative database that consolidates data from the VA electronic medical record. Data were collected between 2014 and 2016.

### Study 1: Access

#### Design

This study, referred to as “Access,” was the initial component of a multi-site, mixed methods study designed to develop a self-report measure of perceived access to mental health care for Veterans. Participants were recruited to take part in a semi-structured qualitative interview, results from which informed the composition of a quantitative measure to assess perceived access to mental health services.

#### Population

To be eligible to participate in this study, participants needed to be U.S. military Veterans between the ages of 18 and 70 years. They needed to have had at least one positive screen for PTSD, alcohol use disorder, or depression documented in their VA medical record in the previous year. We excluded Veterans with documented psychosis or dementia due to concerns that these conditions could limit the recall and cognitive function necessary to complete a meaningful qualitative interview. To ensure geographic diversity within our sample, we recruited participants from three separate clinics within each of three Veterans Integrated Service Networks (VISNs; VISN 1 in the Northeast, VISN 16 in the Central South, and VISN 21 in the West). The total population of Veterans meeting inclusion criteria for the study was 3,461.

#### Recruitment procedures

Using CDW-generated lists of potentially eligible Veterans, we mailed successive waves of opt-out packets, in batches of 30–60 packets each, to Veterans within each of the three study VISNs. Successive waves of opt-out packets for each study VISN were not mailed simultaneously, but were instead mailed when study staff were nearing the end of the previous wave’s call list for that VISN. Opt-out packets included a letter briefly describing the study, and stating that recipients could receive a call from the study team unless they opted-out by returning an enclosed, self-addressed and stamped response form. See Additional file 1 for the text of the opt-out letter. Potential participants also were able to opt-out by calling a toll-free phone number included in the mailing. Those who did not opt out within two weeks were called by trained study staff to discuss study participation. Our study protocol allowed us to leave up to three unanswered messages (or make up to five calls when it was not possible to leave a message) before outreach efforts ceased. When eligible Veterans called back and left messages for study staff indicating interest in the study, the protocol allowed staff to make additional calls beyond the limits mentioned above. Once study staff reached potential participants, they explained the study in detail, assessed interest, and confirmed eligibility.

Recipients for each wave of opt-out packets were selected to ensure enrollment from specific sub-populations in each VISN: women, members of racial/ethnic minorities, a balance of rural and urban residents, and a balance of Veterans with and without a history of mental health service use. For example, some waves of opt-out packets were mailed exclusively to female Veterans to attain our target of 25% female participants in the final sample (while the population served by VA is about 10% female). Some waves were mailed exclusively to Veterans below age 40 to attain our target of 50% Veterans below age 40. In total, we mailed 230 opt-out packets to Veterans in VISN 1 over six waves, 140 packets to Veterans in VISN 16 over three waves, and 215 packets to Veterans in VISN 21 over six waves. Our target sample size was 90 Veterans (30 per VISN), but our protocol allowed recruitment efforts to cease if saturation was achieved—that is, if additional interviews added no new information or topics [25].

#### Institutional review board (IRB) issues

As a multi-site study, Access sought approval from the VA Central IRB. Obtaining Central IRB approval for the opt-out recruitment procedures required several steps beyond those we have encountered when using more traditional, clinic-based recruitment methods. First, a Waiver of Health Insurance Portability and Accountability Act (HIPAA) Authorization was obtained to allow the research team to access CDW data needed to identify potentially eligible Veterans and obtain their contact information. Second, because “cold calling” Veterans is not allowed under VA research guidelines, we specified that we would only call potential participants once they had had a minimum of two weeks to return the opt-out response form. Third, to maximize recruitment rates (including recruitment of as diverse a sample across all of our sub-population strata as possible), we wanted to offer potential participants the option of completing the study entirely by phone without an in-person visit [13]. This required obtaining a Waiver of Written Informed Consent to allow us to elicit informed consent verbally over the phone, rather than obtaining written informed consent by mail. The latter is a time-consuming and burdensome process for participants and researchers alike; paradoxically, it also increases the risk for breach of confidentiality due to the possibility that documents containing personal health information may be lost in the mail. Telephone interviews also required that the Waiver of HIPAA Authorization (referenced above) refer not only to access CDW data for recruitment purposes, but also to specifically allow telephone interviews without a signed HIPAA Authorization.

#### Study procedures

While the focus of this manuscript is on the utility of opt-out recruitment methods in applied research settings, we include brief descriptions of study elements to provide context, especially with respect to what potential participants were asked to do. Participants in Access completed a set of tasks during a 1.5 - 2 h session (primarily face-to-face, although sessions for six participants were conducted by telephone). All sessions included obtaining written (for in-person interactions) or verbal (for phone interactions) informed consent and completing a semi-structured qualitative interview regarding factors that can affect access to mental health services by Veterans. All participants also completed a packet of self-report questionnaires (via pen-and-paper for in-person sessions, and verbally for phone sessions). Lastly, each session also involved structured discussions of barriers to seeking mental health care.

#### Research assistant call logs

In addition to the formal data collection described above, research assistants for Access kept call logs with brief notes regarding the reasons provided by potential participants for their interest or disinterest in study participation.

### Study 2: Tailoring

#### Design

The second study (referred to as “Tailoring”) was the quantitative telephone survey component of a mixed methods study designed to assess the influence of rural/urban differences in attitudes toward mental health care on rates of mental health service use. Participants for Tailoring completed an interviewer-administered, structured questionnaire.

#### Population

This study also enrolled U.S. military Veterans aged 18–70 years. To be eligible, Veterans also had to have had at least one CDW-documented positive screen for depression or PTSD between October 2009 and September 2012, and reside in VISN 1 (Northeast), 16 (South Central region), 19 (Rocky Mountain region), or 23 (North Central region). Veterans with a diagnosis of dementia or a psychotic disorder were excluded from the sampling frame. The total number of Veterans meeting inclusion criteria for the study was 70,119.

#### Recruitment procedures

Using the contact data obtained from CDW, we mailed successive waves of opt-out packets to eligible Veterans (approximately 100 every 2 weeks). As with Access, successive waves of opt-out packets for each study VISN were not necessarily mailed simultaneously, but were instead mailed when study staff were nearing the end of the previous wave’s call list for that VISN. Opt-out packets included a letter explaining the study and informing recipients that they would receive a call from study personnel unless they opted out, either by calling the indicated toll-free number or by mailing back the opt-out response form in the stamped, addressed envelope included in the packet. The packet also included blank copies of informed consent and HIPAA authorization forms for potential participants’ consideration. See Additional file 2 for the text of the opt-out letter for Tailoring.

We used stratified sampling to select Veterans from the sampling frame to receive recruitment letters. We aimed to recruit Veterans from a variety of distances from the Veteran’s residence to the nearest VAMC (evenly distributed among ≤15 miles, 16–49 miles, ≥50 miles). We aimed to have 25% of our final sample be female (while the population served by VA is about 10% female). As with Access, we determined the recipients of later waves of opt-out packets based on these target numbers (e.g. by sending later waves to only female Veterans, or only Veterans living a certain distance from their nearest VAMC, as needed).

Two weeks after the mailing date, trained interviewers began calling those Veterans who had not opted-out. Up to nine calls (three during working hours, three during weekday evening hours, three on weekends) were made over at least a two-week period to each telephone number available in CDW. As with the Access study, additional calls could be made to potential participants who left a voicemail message for study staff indicating interest. Once an interviewer reached a potential participant, the interviewer described the study purpose and procedures in detail, assessed interest and, if appropriate, verified the Veteran’s eligibility. A total of 3,703 opt-out packets were mailed to Veterans in 42 waves (a total of 849–1,041 packets per VISN), with a target sample size of 750 participants.

#### Institutional review board issues

Tailoring obtained a Waiver of Informed Consent and Waiver of HIPAA Authorization from the Central Arkansas Veterans Healthcare System IRB to permit identification of eligible Veterans from CDW data and to obtain their contact information (name, address[es] and telephone number[s]). Tailoring also received a Waiver of Written Informed Consent for telephone survey participation. Trained interviewers verbally reviewed a HIPAA Authorization form and the elements of informed consent with participants prior to starting the telephone survey. The review of the elements of informed consent culminated in study staff obtaining verbal informed consent from all participants.

#### Study procedures

Following informed consent, interviewers administered a structured questionnaire that covered individual characteristics, individual attitudes and beliefs about mental health care, and patterns and pathways of mental health service use (if any).

### Statistical analysis

Analyses were performed for each study separately. We used independent samples *t*-tests, Fisher’s exact tests, and chi-squared tests to compare the characteristics of participants in a study to those of (a) all Veterans who met the study’s inclusion criteria, (b) all Veterans who were mailed an opt-out packet for the study, and (c) all Veterans mailed an opt-out packet, excluding those whose packet was marked as “return to sender” by the postal service due to an incorrect address. The variables on which these groups were compared included age, gender, race/ethnicity, past history of mental health service use, and rurality, all drawn from administrative data in the CDW. To examine the resource demands of opt-out approaches, we also calculated the average number of opt-out packets and the average number of calls required to obtain one study participant. Substantive findings from the Access and Tailoring studies will be reported elsewhere.

## Results

### Recruitment rates

**Fig. 1:**
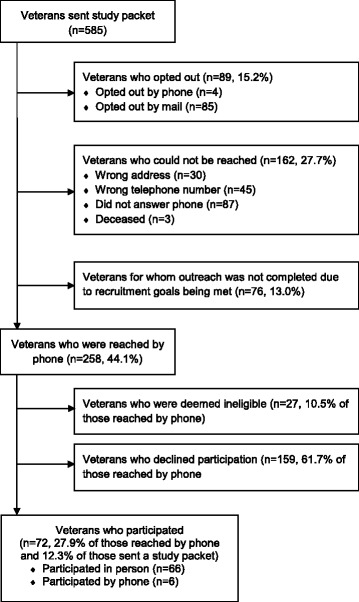
Access study flow diagram

**Fig. 2:**
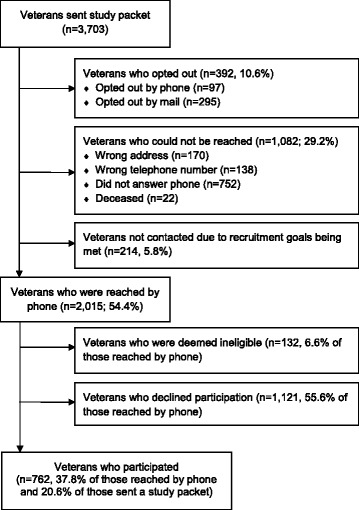
Tailoring study flow diagram

Figures 1 and 2 are recruitment flow diagrams for the Access and Tailoring studies. Relatively few potential participants who received opt-out packets chose to opt out of the study prior to phone contact from staff: 15.2% (Access) and 10.6% (Tailoring). An additional 27.7% (Access) and 29.2% (Tailoring) of potential participants could not be reached due either to incorrect address information (in which case attempting phone contact would have involved “cold calling” because study packets had not been delivered) or unsuccessful phone contact despite multiple attempts. Among those potential participants who could be reached by phone, 27.9% (Access) and 37.8% (Tailoring) agreed to participate.

### Comparisons of the participant samples

Table 1 shows key demographics for all potential Access study participants (*n* = 3,461), for those who were mailed opt-out packets (*n* = 585), for those who received opt-out packets (i.e., packets were not returned to sender; *n* = 557), and for the final sample of Veterans who participated after undergoing the opt-out recruitment process (*n* = 72). Table 2 presents corresponding information for the Tailoring study (n’s of 70,119; 3,703; 3,533 and 762, respectively).

**Table 1:** Representativeness of participant sample (Access study)

		Veterans eligible to receive opt-out letter	Veterans mailed opt-out letter	Veterans who received opt-out letter	Veterans who participated	*p*-value (eligible vs. participated)*	*p*-value (mailed opt-out vs. participated)	*p*-value (received opt-out vs. participated)
*N*		3461	585	557	72			
		Mean (SD)	Mean (SD)	Mean (SD)	Mean (SD)	*p*	*p*	*p*
Age	(Years)	52.6 (13.4)	48.0 (15.0)	48.2 (15.1)	44.7 (14.0)	<.0001	.07	.06
		*N* (%)	*N* (%)	*N* (%)	*N* (%)	*p*	*p*	*p*
Gender	Male	3258 (94.1)	483 (82.6)	465 (83.5)	55 (76.4)	<.0001	.20	.14
	Female	203 (5.9)	102 (17.4)	92 (16.5)	17 (23.6)			
Race	Native American	26 (0.8)	26 (4.4)	23 (4.1)	2 (2.8)	<.0001	.75	.76
	Asian/Pacific Islander	61 (1.8)	61 (10.4)	61 (11.0)	7 (9.7)			
	Black	850 (24.6)	101 (17.3)	91 (16.3)	13 (18.1)			
	White	2161 (62.4)	369 (63.1)	356 (63.9)	45 (62.5)			
	Unknown	412 (11.9)	66 (11.3)	64 (11.5)	12 (16.7)			
Ethnicity	Hispanic	55 (1.6)	55 (9.4)	51 (9.2)	9 (12.5)	<.0001	.40	.37
	Not Hispanic	3117 (90.1)	481 (82.2)	458 (82.2)	57 (79.2)			
	Unknown	289 (8.4)	49 (8.4)	48 (8.6)	6 (8.3)			
MH Use	MH Use	1671 (48.3)	277 (47.4)	262 (47.0)	46 (63.9)	.01	.01	.01
	No MH Use	1790 (51.7)	308 (52.7)	295 (53.0)	26 (36.1)			
Rural/Urban	Rural	1191 (34.4)	236 (40.3)	229 (41.1)	32 (44.4)	.09	.45	.59
	Urban	2239 (64.7)	349 (59.7)	328 (58.9)	40 (55.6)			
	Missing	31 (0.9)	0	0	0			

*Note that *p*-values are calculated from independent samples t-tests for continuous variables and chi squared tests for categorical variables

**Table 2:** Representativeness of participant sample (Tailoring study)

		Veterans eligible to receive opt-out letter	Veterans mailed opt-out letter	Veterans who received opt-out letter	Veterans who participated	*p*-value (eligible vs. participated)*	*p*-value (mailed opt-out vs. participated)	*p*-value (received opt-out vs. participated)
*N*		70119	3703	3533	762			
		Mean (SD)	Mean (SD)	Mean (SD)	Mean (SD)	*p*	*p*	*p*
Age	(Years)	51.6 (13.8)	50.8 (13.8)	51.0 (13.8)	51.6 (12.5)	.35	.48	.70
		*N* (%)	*N* (%)	*N* (%)	*N* (%)	*p*	*p*	*p*
Gender	Male	62280 (88.8)	2947 (79.6)	2806 (79.4)	574 (75.3)	<.0001	<.01	.01
	Female	7839 (11.2)	756 (20.4)	727 (20.6)	188 (24.7)			
Race	Native American	1488 (2.1)	66 (1.8)	64 (1.8)	13 (1.7)	.08	.82	.86
	Asian/Pacific Islander	833 (1.2)	42 (1.1)	40 (1.1)	8 (1.1)			
	Black	12170 (17.4)	474 (12.8)	451 (12.8)	105 (13.8)			
	White	50271 (71.7)	2811 (75.9)	2688 (76.1)	582 (76.4)			
	Unknown	5357 (7.6)	310 (8.4)	290 (8.2)	54 (7.1)			
Ethnicity	Hispanic	n/a	n/a	n/a	46 (6.0)	n/a	n/a	n/a
	Not Hispanic	n/a	n/a	n/a	712 (93.4)			
	Unknown	n/a	n/a	n/a	4 (0.5)			
MH Use	MH Use	46672 (66.6)	2498 (67.5)	2375 (67.2)	532 (69.8)	.06	.20	.17
	No MH Use	23447 (33.4)	1205 (32.5)	1158 (32.8)	230 (30.2)			
Rural/Urban	Rural	20426 (29.1)	1070 (28.9)	1023 (29.0)	230 (30.2)	.39	.45	.46
	Urban	49279 (70.3)	2611 (70.5)	2489 (70.5)	525 (68.9)			
	Missing	414 (0.6)	22 (0.6)	21 (0.6)	7 (0.9)			

*Note that *p*-values are calculated from independent samples t-tests for continuous variables and chi squared tests for categorical variables

While Veterans who participated in the Access study differed from the overall population of eligible Veterans on many variables, this was expected because we had intentionally oversampled specific groups (e.g. female Veterans, racial/ethnic minority Veterans). Data in Table 1 indicate that the final sample of participants from the Access study was did not differ significantly from those who were mailed opt-out packets on age, gender, race, Hispanic ethnicity or residence (rural/urban). They did differ significantly on use of mental health care: participants were more likely to have used mental health services than the pool of Veterans receiving opt-out packets (64 vs. 47%, *p* = .01). The only statistically significant differences in any of the comparisons for the Tailoring study (Table 2) reflect the purposeful oversampling of women.

### Resource demands — packets sent per participant recruited

As shown in Figs. 1 and 2, a minority of potential participants who were sent opt-out invitation packets ultimately participated in the studies: 12.3% of potential Access participants (72/585) and 20.6% of potential Tailoring participants (762/3,703). A small proportion of that is due to packets having been sent out but not fully followed-up because recruitment efforts ceased once enrollment goals were achieved (76 packets [13%] for Access; 214 packets [5.8%] for Tailoring). For Access, the number of opt-out packets sent per participant recruited ranged from 6.7 to 9.2 packets per participant across its three study VISNs (chi squared = 1.47, df = 2, *p* = .48). For Tailoring, they ranged from 4.3 to 5.7 packets per participant across four study VISNs (chi squared = 6.11, df = 3, *p* = .11).

The opt-out packets for Access weighed 1.7 oz, while those for Tailoring weighed 2.3 oz. Postage costs per opt-out packet for Access were approximately $0.90 U.S. (one stamp for the mailing itself, and one additional stamp inside on the self-addressed opt-out card so that respondents could opt out free of charge). Those for the heavier Tailoring packet were approximately $1.35 U.S. (two stamps for the packet itself, and one for the enclosed self-addressed opt-out card).

### Resource demands — calls required to recruit one participant

On average, 2.9 ± 1.9 (SD) phone calls were needed to arrive at a final status (i.e., interview scheduled, Veteran declined to participate, or study staff abandoned unsuccessful outreach efforts) for Veterans we attempted to contact for the Access study. Veterans who participated in the study averaged more phone calls from study staff (4.5 ± 1.8), due primarily to the additional calls needed to schedule the study session itself after initial contact was made. For Tailoring, on average, 6.1 ± 5.1 calls were needed per Veteran to arrive at a final status; an average of 4.7 ± 3.7 calls were needed for the subset of Veterans who participated in the study. The larger number of calls required overall in Tailoring is a result of the more intensive calling procedures followed in that study (i.e., up to nine calls to each of the Veteran’s available telephone numbers) before efforts to contact the potential participant were suspended).

Study staff estimated that calls to potential participants resulting in no answer or a voicemail took, on average, 1 minute each. Calls that involved some discussion with potential participants, but did not result in consent, took about 5 minutes on average across both studies. Calls that involved consent to participate in the study took about 15 minutes for Access, and about 7 minutes for Tailoring. The extra time needed for consent calls for Access compared to Tailoring was due, in part, to the need to coordinate in-person participation for Access (including describing the physical address and check-in procedures for the clinic). Based on these estimates, it took about 40 h of phone time to recruit 72 Access participants, and about 500 h to recruit 762 Tailoring participants. These estimates include only time on the telephone itself, but not other tasks associated with calling (e.g. looking up phone numbers or documenting phone contact).

### Research assistant call logs

Informal notes taken by research assistants during the recruitment process of the Access study indicated two broad trends related to study interest and participation. First, these notes indicated that many participants endorsed either very good or very bad experiences using VA treatment. Second, several Veterans noted that the potential to improve healthcare delivery to other Veterans was a strong motivator for them to participate in the study.

## Discussion

In this paper, we describe successful recruitment efforts using opt-out methods drawing potential samples from administrative databases in two studies of access to mental health care among Veterans.

### Recruitment rates

Both the Access and Tailoring studies achieved their target sample sizes. Studies with a prominent qualitative component, like Access, continue enrollment until saturation is achieved, i.e., until additional interviews add no new information and no new topics emerge [25]. Thus, when saturation had been achieved with 80 participants (72 of whom had been recruited via opt-out procedures), Access suspended recruitment. Tailoring interviewers enrolled 762 Veterans, exceeding its target of 750. Enrollment (as a percentage of opt-out letters sent) differed by study, with about 12% of opt-out letters for Access leading to research participation and nearly double that rate (21%) for Tailoring. This difference in recruitment rates was likely driven, at least in part, by differences in the nature of participation and sampling in the two studies. Most participants in the Access study needed to present to a VA clinic for a face-to-face research session, while participation in Tailoring took place completely by telephone: the former places a substantially greater burden on potential participants. Another likely explanation for this finding is that Tailoring interviewers were required to make more calls to each available telephone number before suspending efforts in this study than was the case for Access interviewers.

Neither Access nor Tailoring included an opt-in comparison arm, as both studies were funded to study Veterans’ experiences with mental health care rather than recruitment methods per se. Unfortunately, opt-in studies of military Veterans to which to compare our results are scarce, and the few we could find focused on biobank research (e.g. [14]) or involved enriched samples that had already participated in previous studies (e.g. [26]). Opt-out response rates for the Access and Tailoring studies can also be compared to published rates from national surveys that recruited via random digit dialing surveys. Past surveys addressed sensitive topics such as sexual violence [27], alcohol use and gambling [28], racial discrimination and workplace harassment by gender [29], pharmacogenetic test result preferences [30], and care coordination for the chronically ill [31]. Response rates for these studies varied dramatically, from 27.6% [31] to 65.4% [28]. Variation in response rates may reflect differences in the sensitivity of the topic covered by the survey, the length of the telephone survey commitment, and the intensity with which the protocol allowed recruiters to pursue responses. For example, one study on workplace harassment study allowed twenty contact attempts, and two calls from interviewers for refusal conversion in the case of a refusal [29]. Regardless of response rates, for ethical reasons, the VA does not permit investigators to use cold calling strategies like random digit dialing.

While the recent literature on non-Veteran samples consistently reports higher rates of recruitment using opt-out rather than opt-in methods, [2, 11–18, 32–36] actual recruitment rates achieved with opt-out methods also varied widely, from lows of about 15% [32, 33] to highs above 70% [35, 36]. Some of the variation undoubtedly arises from differences in what participants are being asked to do. For example, the highest recruitment rate (83%) was reported by Vellinga and colleagues [36] who simply asked patients in Irish general practices for permission to review their medical records as part of a study of urinary tract infections. In this case, completing the study required no effort from participants other than providing consent. Studies focused on prevention or treatment of specific clinical conditions such as angina [11] or colorectal cancer [34] from which participants might reasonably expect clinical benefit also reported recruitment rates at the higher end of the range (50 and 67%, respectively). On the other hand, observational studies with a non-clinical focus such as quality of end-of-life care [12] or road safety research [32] reported lower recruitment rates (40 and 17%, respectively).

Given the non-clinical focus of the Access and Tailoring studies, it is not surprising that our recruitment rates of 12 and 21% were at the lower end of rates reported for opt-out recruitment. Further, both studies required 1.5–2 h of active participation in-person or by phone, putting a heavier demand on participants than was the case for many of the studies found in the literature.

### Comparisons of the participant samples

Both Access and Tailoring were generally successful in recruiting the samples they had targeted. The Access study purposefully oversampled women and racial/ethnic minorities, and endeavored to recruit 50% rural residents. The oversampling of female Veterans (who are younger, on average, than male Veterans) contributed to a disproportionately younger sample compared to the overall population of potentially eligible Veterans. The participant sample for Access also had a significantly higher rate of past-year mental health service utilization than did the population of Veterans who were mailed opt-out packets (64 vs. 47%). This pattern was seen in Tailoring as well, although the difference was smaller and not statistically significant. It is plausible that several factors associated with non-participation in mental health treatment might also be associated with non-participation in mental health research, including physical disability, transportation barriers, paranoia, or other mental health symptoms. Thus, findings from Access and Tailoring are generally consistent with previous reports that opt-out methods generate samples with characteristics that reasonably (but not completely) mirror those in their reference populations [11, 13, 36].

### Resource demands

Among the strengths of this effort is the practical information it affords regarding the resource demands of opt-out recruitment. For Access, it took between 6.7 and 9.2 mailed opt-out packets to recruit one study participant across the study’s three VISNs. For Tailoring, it took between 4.3 and 5.7 packets to recruit one participant across four study VISNs. The differences among VISN’s did not reach statistical significance in either study.

The average number of phone calls required to reach a final status for each participant ranged from about 3–6 for our two studies. For Access, arriving at a final status required more calls for eventual participants, due to the additional calls required to schedule in-person study participation. In contrast, for Tailoring, non-participants required more calls before final status was achieved; this is likely to reflect the larger number of calls that were allowed under the Tailoring protocol. Across the two studies, it took about 30–40 min of phone time to successfully recruit one participant. Taken together, these findings emphasize the critical importance of seemingly small design decisions (such as the maximum number of calls allowed to potential participants before outreach efforts cease) in determining how much effort is spent on different recruitment activities. It is our hope that other researchers will find the information on recruitment design decisions for Access and Tailoring (and the corresponding resource demands and recruitment outcomes) useful in informing the design of their future studies.

### Possible reasons for participation

While neither Access nor Tailoring explicitly queried Veterans regarding their reasons for participating, informal comments from Access participants suggested two contributing factors. First, several Veterans said that they were excited to participate because they had either had very good or very bad experiences accessing VA mental health care, and this study was an opportunity to have their experience be heard. This is consistent with previous literature that has found personal salience to be a key factor contributing to study participation [24, 37]. Second, other Veterans noted that participating was a way for them to “give back” to VA and possibly improve the mental health services that their fellow Veterans might need. This strong orientation toward service and community participation has also been the focus of previous research on study participation [24, 38, 39]. Such altruism may be especially strong in a Veteran population with its sense of camaraderie, but may not generalize to other populations.

### Limitations

Several limitations should be taken into account when considering study findings, especially for other researchers who are considering using similar methods in their own studies. First, there are several challenges to the generalizability of findings to other populations and settings. Both studies recruited Veterans already in touch with the VA health care system, and both depended on administrative data to generate a sampling frame. Response rates and resource demands could be higher for studies attempting to recruit Veterans eligible for but not using VA care. It would be reasonable to expect that such Veterans would be even less likely to participate in a study on access to mental health services than those who have received at least some VA health care. In addition, our results may be most applicable to other large health systems with administrative databases similar to those in the VA (e.g. Kaiser Permanente). Second, neither Access nor Tailoring included formal evaluations of why Veterans chose to participate or not participate in the study—although we do report brief findings from informal call notes. Third, our studies were not designed to compare recruitment rates or resource demands across recruitment strategies. Thus, while our results will be useful to others in designing, budgeting, and executing studies employing opt-out recruitment strategies, they do not shed light on the relative utility of opt-out versus other strategies.

## Conclusions

Studies examining access to care must focus carefully on recruitment and design issues, lest researchers unwittingly fail to sample those people who never or rarely access care. The two studies described in this paper used opt-out strategies to achieve ultimate recruitment rates of 12 and 21% of those sent an opt-out packet (for in-person and telephone-based participation, respectively). While the final samples of participants were broadly similar to those who were mailed opt-out packets, in the Access study, opt-out alone was not sufficient to eliminate non-response bias reflected in higher rates of mental health service use among study participants. The sampling frame generated for Tailoring, which took history of service use into account, was able to reduce that problem substantially. This suggests that future studies of mental health access may want to focus outreach efforts specifically toward potential participants who have not used the services in question and, when sampling frames can be constructed, use sampling strategies that increase the likelihood of recruiting such participants.

Our opt-out recruitment efforts required a relatively high number of calls per potential participant. Had we used opt-in methods instead, it is plausible that recruitment would have required fewer calls, but at the expense of sending many more recruitment packages to potential participants. Researchers will need to balance these considerations when designing studies investigating access to care that rely on administrative databases for potential selection of participants.

## Additional files

Additional file 1: Opt-Out Letter (Access study). (DOCX 24 kb)

Additional file 2: Opt-Out Letter (Tailoring study). (DOCX 15 kb)

## Abbreviations

CDW: Corporate Data Warehouse
HIPAA: Health Insurance Portability and Accountability Act
IRB: Institutional review board
PTSD: Posttraumatic stress disorder
VA: Department of Veterans Affairs
VISN: Veterans Integrated Service Network
