# Supplementary material for: Practical application of opt-out recruitment methods in two health services research studies
Source: BMC Med Res Methodol. 2017 Apr 14;17:57. doi: 10.1186/s12874-017-0333-5 (PMC5391553; doi:10.1186/s12874-017-0333-5)
Supplement: Supplementary file 1 — Opt-Out Letter (Access study). (DOCX 24 kb) [file 12874_2017_333_MOESM1_ESM.docx]

< Today’s Date >

< Veteran’s Name >

< Address >

< City, State, Zip >

RE: Development and Validation of a Perceived Access Measure

Dear Veteran Name,

I would like to let you know about a study being offered at the Central Arkansas Veterans Healthcare System. The purpose of this study is to develop a questionnaire about how to improve access to mental health care for veterans. We want to learn about things that get in the way of receiving mental health services at VA mental health clinics. This study is designed for Veterans who might want help for mental health problems.

If you decide to participate, you will be asked to take part in one in-person or telephone interview that will last approximately 1½ – 2 hours. If you are eligible for the study, you will answer some general questions about yourself (age, gender, etc.), your current mental health symptoms, and barriers to receiving mental health care. You will also answer questions about your experiences accessing mental health care at the VA. We will also record information from your medical record about things like your diagnoses. If you agree, this interview will be recorded. You will receive a $30 gift card from a VA-approved merchant for completing the interview.

If you **DO NOT** want to be contacted about this study:

**Please contact research staff at 501-555-1084 or 501-555-1730. If you are calling long distance, please call 1-800-555-9148.**

**OR**

**Complete the enclosed response form and use the pre-paid self-addressed envelope to mail the form back.**

If you do not return the response form or call the telephone number in two weeks, a research assistant may call you to describe the study in more detail and answer any questions you may have. You will be under no obligation to participate in the study at this point. If you do decide to participate, we will make arrangements for the interview at that time. If you change your mind later, you will be free to end your participation at any time.

If you are interested in learning more about the study or have any questions, please call one of the telephone numbers listed above to speak with a member of the research team.

Your participation in this study will help the VA improve veterans’ access to high quality mental health care.

Thanks for your consideration,

< PI Signature >

Jeffrey M. Pyne, MD

Principal Investigator

## DO NOT CONTACT ME RESPONSE FORM

***Development and Validation of a Perceived Access Measure***

If you **DO NOT** want to be contacted about this study, please use this form to let us know. Print your name, sign it, and then send it back to us. Or you may call the phone number below to request that we not contact you. If we don’t hear from you, you may receive a telephone call from a Re­search Assistant in about two weeks. The Research Assistant will explain the study in more detail and answer any questions you may have.

**You are under no obligation to participate.**

**I DO NOT want to be contacted about this study.**

My name: _______________________________

My signature: ____________________________

**Please mail this form in the enclosed postage-paid envelope.**

If you like, you may call and let us know
you do not want to be contacted at**:**

**501-257-1084 or 501-257-1730**

**If you are calling long distance, please call 1-800-250-9148.**

|  |  |  |
| --- | --- | --- |
