# Supplementary material for: Practical application of opt-out recruitment methods in two health services research studies
Source: BMC Med Res Methodol. 2017 Apr 14;17:57. doi: 10.1186/s12874-017-0333-5 (PMC5391553; doi:10.1186/s12874-017-0333-5)
Supplement: Supplementary file 2 — Opt-Out Letter (Tailoring study). (DOCX 15 kb) [file 12874_2017_333_MOESM2_ESM.docx]

< Today’s Date >

< Veteran’s Name >

< Address >

< City, State, Zip >

Dear <Veteran’s Name >,

I would like to invite you to take part in a VA research study being conducted by the Central Arkansas Veterans Healthcare System.

The study is called “Tailoring Interventions for Rural Veterans: What We Need to Know.” The purpose of the study is to help the VA improve access to services for Veterans by better understanding their attitudes toward treatment for physical, mental and emotional problems. Veterans do NOT have to be using VA healthcare services currently or have experienced a physical, mental or emotional problem to help us with this study.

If you are eligible and choose to participate in the study, you will take part in a telephone interview that will last about 1-1.5 hours. An interviewer will ask you questions about yourself, your use of healthcare services, and your thoughts about healthcare and using healthcare services, especially mental healthcare services. We have enclosed copies of the study informed consent forms which give more details about it. If you complete an interview, you will receive a check for $40.

If you **DO NOT** want to be contacted about this study, please let us know. You can do that by:

**Calling Ms. Mary Kate Bartnik, our Project Coordinator, at 501-555-1702 or toll-free at 1-888-555-1978**

**OR**

**Completing the enclosed form and returning it to us in the enclosed stamped, addressed envelope.**

If we do not hear from you by phone or mail, a research assistant will call you about two weeks after the date of this letter. The call will come from area code 501. If you have caller ID, “US Government” may appear on the display screen.

When the research assistant calls, he or she will explain the study in more detail and answer any questions you may have. You are under no obligation to participate.

The telephone number we have for you is: < Veteran’s Phone # >. If this number is not correct or you would like us to use a different number, please let us know. You can do that either by calling Ms. Bartnik at one of the numbers above or by correcting the number on the enclosed form and mailing the form back to us.

If you would like to verify the legitimacy of the study with someone not directly related to it, you may call the Central Arkansas Veterans Healthcare System Institutional Review Board (IRB) at (501) 555-6521.

Thank you for your consideration.

Sincerely,

< PI Signature >

Ellen P. Fischer, PhD

Principal Investigator
